# Chemical State Mapping of Degraded B_4_C Control Rod Investigated with Soft X-ray Emission Spectrometer in Electron Probe Micro-analysis

**DOI:** 10.1038/srep25700

**Published:** 2016-05-10

**Authors:** R. Kasada, Y. Ha, T. Higuchi, K. Sakamoto

**Affiliations:** 1 Institute of Advanced Energy, Kyoto University, Gokasho, Uji, Kyoto 611-0011, Japan; 2 Nippon Nuclear Fuel Development, Co., Ltd., 2163 Narita-cho, Oarai, Ibaraki 311–1313, Japan

## Abstract

B_4_C is widely used as control rods in light water reactors, such as the Fukushima Daiichi nuclear power plant, because it shows excellent neutron absorption and has a high melting point. However, B_4_C can melt at lower temperatures owing to eutectic interactions with stainless steel and can even evaporate by reacting with high-temperature steam under severe accident conditions. To reduce the risk of recriticality, a precise understanding of the location and chemical state of B in the melt core is necessary. Here we show that a novel soft X-ray emission spectrometer in electron probe microanalysis can help to obtain a chemical state map of B in a modeled control rod after a high-temperature steam oxidation test.

Control rods used in boiled-water reactors (BWRs) in Japan, including all units at the Fukushima Daiichi nuclear power plant (NPP), consist of stainless steel tubes filled with B_4_C granules^1^. B_4_C is stable at the normal NPP operating temperature of ~300 °C, as it melts at 2450 °C^2^. However, the eutectic interaction of B_4_C with the adjacent stainless steel leads to its liquefaction at temperatures exceeding 1200 °C under severe accidental conditions^1^,^2^,^3^,^4^,^5^. The appearance of this liquid phase causes the phenomenon of candling and relocation, in which the liquid phase sinks to the bottom of the nuclear core. The proper pouring of cooling water is necessary to prevent achieving recriticality when B is not available as a neutron absorber at the upper part of the core. In addition, the oxidation of B_4_C by high-temperature steam may first form B_2_O_3_ and then gaseous boric acids (HBO_2_ and H_3_BO_4_)^3^. The release of these gases may affect the source term of radioactive materials. Therefore, the prediction and evaluation of B and the chemical state thereof is necessary to control accidents.

The spatial distribution of B in B_4_C after severe accidental simulation tests has been analyzed mainly by scanning electron microscopy (SEM) with energy-dispersive X-ray spectroscopy (EDS) and by electron probe microanalysis (EPMA) with wavelength-dispersive X-ray spectroscopy (WDS)^1^,^5^. However, these conventional methods do not easily provide chemical state information, as they are limited by insufficient energy resolution. Although soft X-ray emission or absorption spectrometry, using synchrotron radiation facilities, may provide an understanding of the chemical state of B^6^,^7^, it is not economically viable to simultaneously obtain a detailed spatial distribution.

In this study, we demonstrate the chemical state mapping of B in a modeled B_4_C control rod after a high-temperature steam oxidation test using the newly developed EPMA-soft X-ray emission spectrometer (SXES) with ultra-high energy resolutions^8^,^9^. EPMA-SXES is a type of WDS that performs a parallel collection of soft X-ray spectra by a combination of an aberration-corrected grating system and a high-sensitivity X-ray charge-coupled device (CCD). Therefore, EPMA-SXES permits the spectral mapping of light elements.

## Results and Discussion

**Figure 1:**
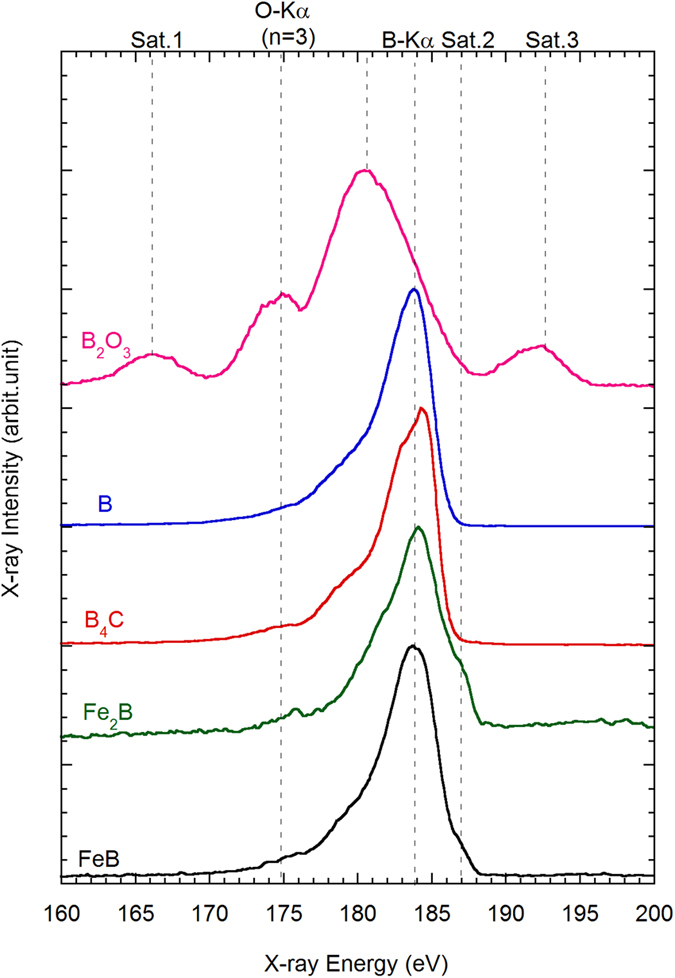
Soft X-ray emission spectra from B_2_O_3_, B, B_4_C, Fe_2_B, and FeB, obtained by EPMA-SXES.

Figure 1 shows the soft X-ray emission spectra of B-Kα from B-containing crystalline materials such as (a) B_2_O_3_, (b) B, (c) B_4_C, (d) Fe_2_B, and (e) FeB as references for EPMA-SXES. The spectra were normalized by the maximum intensity of each B-Kα peak. As reported in the Handbook of X-Ray Data^10^, the B-Kα peak is observed at 183.5 eV for B. The B-Kα peak of B_4_C shifts to a slightly higher energy at 184 eV and shows a characteristic shape with decreased intensity at lower-energy. The change in the spectral shape is attributed to the bonding of B and C within B_4_C. The spectra of the iron borides FeB and Fe_2_B depict clear shoulders, designated as Sat.2 in the upper horizontal axis of Fig. 1, near 187 eV. These shoulders relate to the Fermi level of the material, as the electron behavior of B in iron borides is more metallic than that in the pure element^11^. The shoulders may also relate to the slightly higher energy of the peak of Fe_2_B than those of B and FeB. Notably, B_2_O_3_ shows a distinctly different spectral shape compared to that of the other B-containing materials. The B-Kα peak of B_2_O_3_ experiences a large shift to a lower energy at 180.5 eV and the formation of two additional satellite peaks: one higher (Sat.3: 192.5 eV) and one lower (Sat.1: 166 eV). Muramatsu *et al.* revealed that the higher-energy satellite peak originated from resonant X-ray Raman scattering and linked the lower-energy satellite peak to a transition from the B 2*p* and O 2*s* molecular orbitals to a core hole at the B 1*s* orbital^12^. Thus, we consider that EPMA-SXES has a sufficiently high energy resolution to determine the chemical state of B from the changes in the spectral shape of the B-Kα peak.

**Figure 2:**
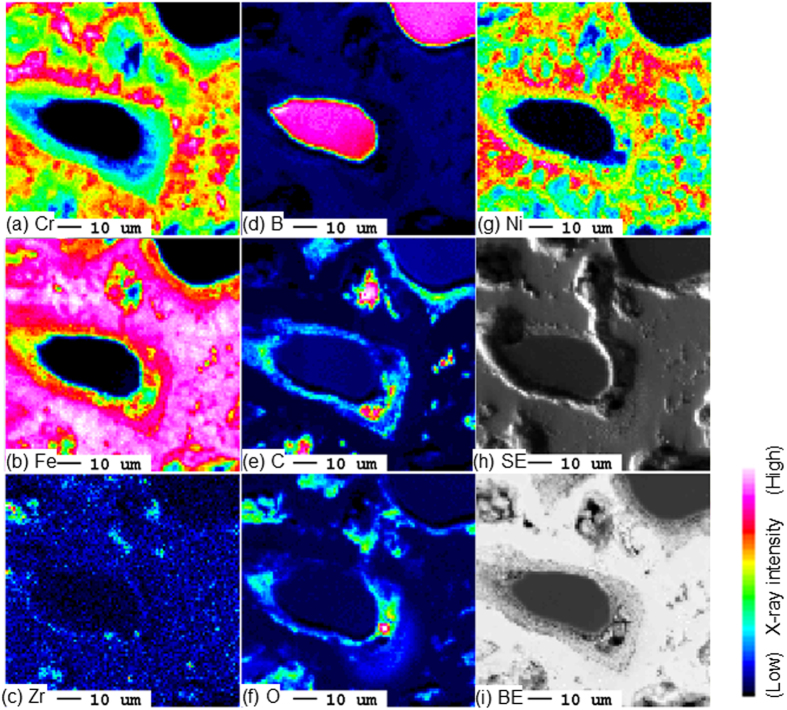
Elemental mapping and scanning electron images obtained by EPMA-WDS on the cross-section of B_4_C control rod after high-temperature steam oxidation test at 1250 °C for 30 min.

Figure 2 shows (a–g) elemental mapping results obtained by conventional EPMA-WDS, (h) a secondary electron image, and (i) a back-scattering electron image on the cross-section of the B_4_C control rod model after exposure to high-temperature steam at 1250 °C for 30 min. Although B_4_C and stainless steel reacted significantly, B_4_C granules remain in the red- and pink-colored region in the (d) B map. Alloying elements of stainless steel such as Fe, Cr, and Ni are observed within the stainless tube where the B_4_C granules were placed before the oxidation test. Because these area also contains some amounts of B, liquefaction by eutectic interactions between B_4_C and stainless steel occurs under the high-temperature oxidation testing condition; the liquid solidifies as the test temperature is decreased. The electron images (h,i) show that the peripheral vicinity of the remaining B_4_C granule comprises a layer of reaction products from B_4_C with stainless steel and/or steam. The elemental maps show that the peripheral vicinity of the remaining B_4_C contains relatively dense O and C. The chemical state information of B will determine whether these are from B_2_O_3_ formed by the oxidation of B_4_C or from other oxides.

**Figure 3:**
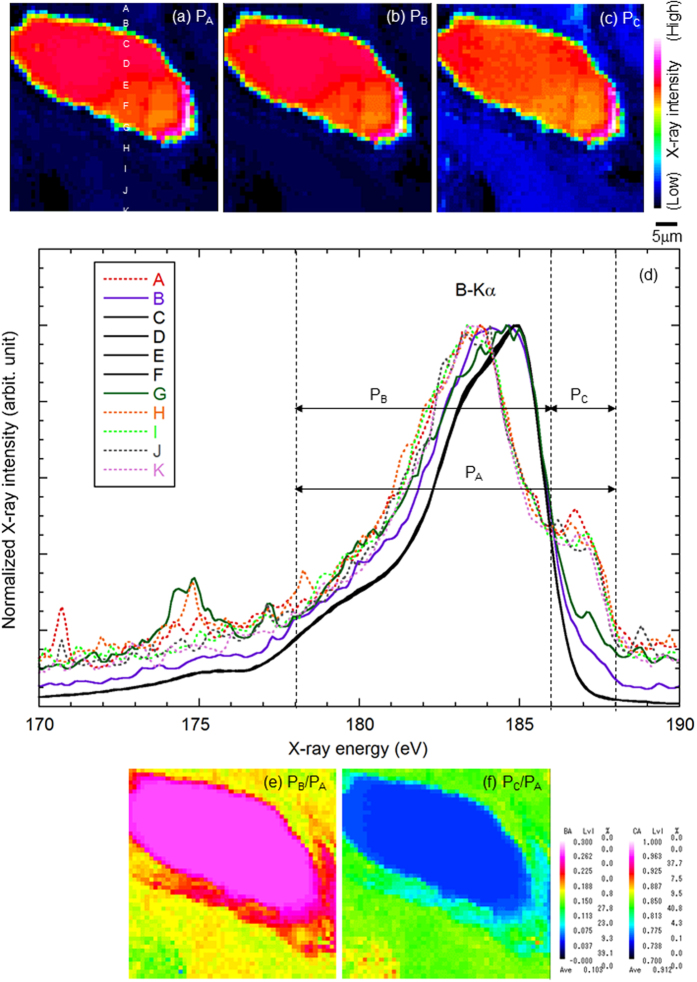
Elemental maps based on (**a**) *P*_A_, (**b**) *P*_B_, and (**c**) *P*_C_ of (**d**) B-Kα spectrum, obtained by EPMA-SXES and re-calculated by peak-area ratio as (**e**) *P*_B_/*P*_A_ and (**f** ) *P*_C_/*P*_A_.

In Fig. 3, we show the EPMA-SXES measurement result of (a–c) a B elemental map for the same position as Fig. 2 and (d) B-Kα spectra from the points A through K, as designated in the elemental map. The B-Kα spectra (solid lines) for the points of the remaining B_4_C granule present the characteristic peak shape of B_4_C, as shown in Fig. 1. However, the B-Kα spectra (dotted lines) for the points farther from the remaining B_4_C granule exhibit a unique shoulder at ~187 eV, similar to the shoulders in the spectra of the iron borides. Thus, EPMA-SXES supports that B in B_4_C reacts with the stainless steel to form borides. Although the peak of O-Kα (n = 3) is also observed near the B_4_C particle, the shape of the B-Kα peak differs from that of B_2_O_3_. Therefore, the O near the B_4_C granule probably results from other metal oxides.

To understand the reaction process of the B_4_C control rod model after high-temperature steam oxidation testing, it is beneficial to visualize the chemical distribution of B in the model. To clarify the chemical state, the elemental maps shown in Fig. 3(a–c) are re-calculated by the areal ratios of the peaks; *P*_A_ is the total area of B-Kα in the range 178–188 eV, *P*_B_ is the area from 178 to 186 eV, and *P*_C_ is the area in 186–188 eV. Figure 3(e,f) show the chemical state maps obtained by calculating *P*_B_/*P*_A_ and *P*_C_/*P*_A_, which display the existing areas of B_4_C and borides separately.

Thus, we succeeded in identifying the chemical state of B and in visualizing the spatial distribution of B with associated chemical states in the degraded B_4_C control rod model, based on EPMA-SXES with ultrahigh energy resolution. Our findings will aid the establishment of severe accident simulation codes and the prediction of the chemical state and physical location of B in the Fukushima Daiichi NPP. In addition, we emphasize that the present lab-scale SXES by EPMA has excellent energy-resolution and higher spatial resolution not only for light elements but also for higher atomic number elements compared with SXES by synchrotron radiation facilities. EPMA-SXES permits the visualization of the chemical state of elements in various materials at the micro-scale.

## Methods

### Reference materials

Five B-containing crystalline materials were prepared; B granules (3–7 mm) and B_2_O_3_ powder were purchased from Kojundo Chemical Laboratory Co., Ltd. FeB and Fe_2_B (500 μm in diameter) were purchased from Goodfellow Cambridge Ltd. B_4_C granules were supplied by a domestic BWR manufacturer in Japan. The chemical compositions were shown elsewhere^7^.

### High-temperature steam oxidation test

**Figure 4:**
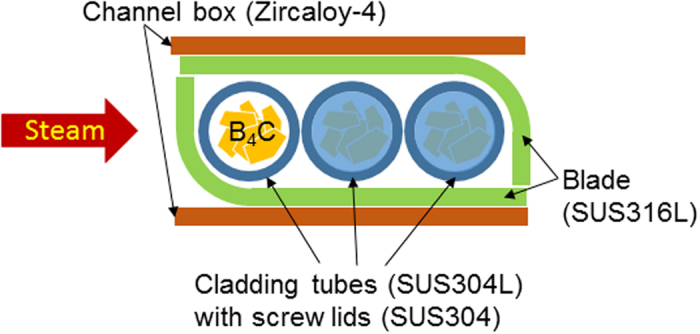
Schematic of the B_4_C control rod model used.

We used a control rod model containing B_4_C granules^7^ as neutron absorbers, SUS304L as cladding tube, SUS316L as control rod blade, and Zircaloy-4 as the channel box material as shown in Fig. 4. The B_4_C granules were placed in the cladding tubes, which are 0.6 mm in thickness, 4.8 mm in outer diameter and 20 mm in length. The vibration method was used for filling B_4_C granules with a packing density of approximately 70%. Then the ends of the tubes were sealed with setscrews of SUS304. Three sets of stainless steel tubes filled with B_4_C were sandwiched by the control rod blade material (SUS316L) and the channel box material (Zircaloy-4), which was coated with an oxide film of approximately 20 μm in thickness on the surface, equivalent to that formed on the actual component by exposure to water vapor at 1000 °C for 10 min.

The high-temperature steam oxidation test was performed on the model for 30 min at 1250 °C, at which point the eutectic interaction of B_4_C and stainless steel is known to be rapid. The heating of the model was performed by a horizontal electric furnace using a KERAMAX (LaCrO_3_) heater. Firstly, the model was set on a sample table located in a low-temperature part of the reaction tube. Secondly, the furnace was kept at the target temperature under flowing humidified Ar gas. Thirdly, the sample table was moved into the high-temperature part of the reaction tube using a heat-resistant push rod. After 30 min in this region, the test was completed by transferring the sample stage, using the push bar, to the opposite low-temperature region. The Ar gas was humidified by bubbling the Ar at 800 cm^3^/min into a constant-temperature water bath kept at 70 °C.

After the high-temperature steam oxidation test, the model was cut at the axial direction center position and embedded into resin. The cross-section was polished with SiC papers and colloidal silica. The final surface of the cross-section was Pt-coated to prevent charge-up.

### EPMA-WDS and SXES

**Figure 5:**
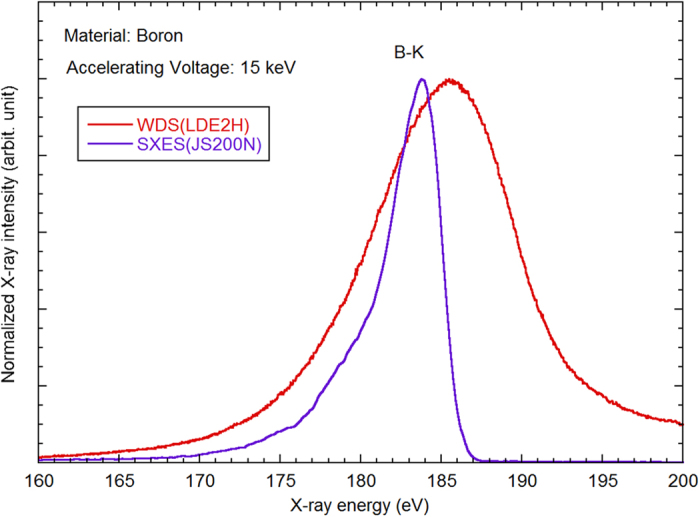
Comparison of B-Kα spectra obtained by EPMA-WDS using LDE2H and EPMA-SXES using JS200N as spectrometer.

The polished sample was examined by a JXA-8500F field emission EPMA (JEOL) equipped with WDS and SXES. The acceleration voltage and beam current of the scanning electron beam were 15 keV and 5.5 × 10^−7^ A, respectively. The energy range of SXES was 54 to 220 eV. The nominal energy resolution for SXES was 0.22 eV. Figure 5 shows the soft X-ray emission spectra of B-Kα from B granules obtained by a conventional WDS (type LDE2H) and the newly developed SXES (type JS200N). The advantages of SXES in energy resolution are evident. The broad asymmetrical peak is characteristic of covalent bonding^11^.

## Additional Information

**How to cite this article**: Kasada, R. *et al.* Chemical State Mapping of Degraded B_4_C Control Rod Investigated with Soft X-ray Emission Spectrometer in Electron Probe Micro-analysis. *Sci. Rep.* **6**, 25700; doi: 10.1038/srep25700 (2016).
